# Characterization of rubella-specific humoral immunity following two doses of MMR vaccine using proteome microarray technology

**DOI:** 10.1371/journal.pone.0188149

**Published:** 2017-11-16

**Authors:** Iana H. Haralambieva, Michael J. Gibson, Richard B. Kennedy, Inna G. Ovsyannikova, Nathaniel D. Warner, Diane E. Grill, Gregory A. Poland

**Affiliations:** 1 Mayo Vaccine Research Group, Mayo Clinic and Foundation, Rochester, MN, United States of America; 2 Division of Biomedical Statistics and Informatics- Department of Health Science Research, Mayo Clinic and Foundation, Rochester, MN, United States of America; Imperial College London, UNITED KINGDOM

## Abstract

**Introduction//Background:**

The lack of standardization of the currently used commercial anti-rubella IgG antibody assays leads to frequent misinterpretation of results for samples with low/equivocal antibody concentration. The use of alternative approaches in rubella serology could add new information leading to a fuller understanding of rubella protective immunity and neutralizing antibody response after vaccination.

**Methods:**

We applied microarray technology to measure antibodies to all rubella virus proteins in 75 high and 75 low rubella virus-specific antibody responders after two MMR vaccine doses. These data were used in multivariate penalized logistic regression modeling of rubella-specific neutralizing antibody response after vaccination.

**Results:**

We measured antibodies to all rubella virus structural proteins (i.e., the glycoproteins E1 and E2 and the capsid C protein) and to the non-structural protein P150. Antibody levels to each of these proteins were: correlated with the neutralizing antibody titer (p<0.006); demonstrated differences between the high and the low antibody responder groups (p<0.008); and were components of the model associated with/predictive of vaccine-induced rubella virus-specific neutralizing antibody titers (misclassification error = 0.2).

**Conclusion:**

Our study supports the use of this new technology, as well as the use of antibody profiles/patterns (rather than single antibody measures) as biomarkers of neutralizing antibody response and correlates of protective immunity in rubella virus serology.

## Introduction

While rubella virus commonly causes mild fever and rash during childhood, serious complications (i.e., miscarriage or birth defects of the fetus/baby, referred to as congenital rubella syndrome/CRS) can arise if infection develops in women during the first months of pregnancy. [1] Rubella virus is able to cross the placenta and replicate in fetal tissues, causing systemic inflammation and resulting in up to a 90% risk of developing CRS depending upon the timing of infection during the pregnancy. [1,2,3] The most common CRS complications include deafness, cataracts and blindness, congenital heart defects, endocrinopathies, microcephaly, encephalopathy, mental retardation, and death. [1,4]

Vaccination programs have drastically reduced the incidence of rubella infection and CRS; however, current estimates suggest that 100,000 cases of CRS still occur worldwide each year. [1] Although endemic rubella transmission has been eliminated in the US, 79 rubella cases and six CRS cases were reported in the US during the 2004–2012 period, primarily in unvaccinated individuals who were infected in other countries. [1,5] Combined with decreasing rates of immunization due to vaccine hesitancy, rubella will remain a public health concern as long as it continues to be endemic or circulate in any area of the world. This points to the necessity of timely and accurate diagnosis of new cases, vaccination of susceptible individuals, monitoring and deeper understanding of vaccine-induced immunity, and the development of newer vaccine candidates.

The rubella virus belongs to the Togaviridae family (genus *Rubivirus*) and is an enveloped, single-stranded RNA virus with a positive polarity genome confined within a capsid that is composed of capsid (C) protein. The genome encodes three structural (C, E1, and E2) and two non-structural proteins. The two surface glycoproteins, E1 and E2 are associated with virus neutralization and protective immunity, while the non-structural proteins (p90 and p150) are considered non-immunogenic. [6] The E1 protein, in particular, is considered to be the immunodominant and hemagglutation-eliciting antigen that predominantly contributes to protective immunity. [7,8,9,10,11,12] Assays such as whole- rubella virus, recombinant protein and synthetic peptide-based enzyme immunoassays (including immunoblot), hemagglutination inhibition assays, and neutralization assays (including a high-throughput immunocolorimetric-based neutralization assay) have been used in large studies for surveillance of rubella vaccine-induced immunity. [13,14,15,16,17,18,19,20] Recent papers and expert reviews from the literature point to the lack of standardization of the international rubella antibody standard and the currently used commercial anti-rubella IgG antibody (Ab) assays (leading to misinterpretation of results), and recommend improved and/or alternative approaches in rubella serology testing (including qualitative testing and/or testing without the use of the existing RUBI-1-94 international rubella Ab standard for calibration of assays). [1,6,21,22,23] Antigen profiling based on a high-throughput microarray technology offers an exciting new opportunity to interrogate the entire viral proteome and assess effective humoral immune responses to vaccination and/or infection. [24,25,26,27] This technology is well suited for dissecting humoral immunity and in-depth understanding of pathogen-specific immune response in systems biology and population genetics studies. [28,29] We have previously used this technology to profile humoral immune responses to the measles component of the MMR vaccine in a cohort of 150 individuals after two vaccine doses, and we have defined a model predictive of measles-specific neutralizing antibody response. [27] In this study, we used a newly developed rubella virus-specific protein microarray chip and probed IgG rubella-specific humoral immune responses in 75 high neutralizing Ab responders and 75 low neutralizing Ab responders (after two MMR vaccine doses) in order to develop rubella vaccine-specific humoral immune profiles (consisting of antibodies to individual rubella virus proteins) associated with neutralization Ab titers and potentially with protective immunity. The results of this study, in concert with previous results, can lead to an enhanced understanding of humoral immunity and immunogenicity of MMR vaccine. These results may also potentially lead to the development of a combined chip for assessment of humoral immunity after MMR vaccination.

## Materials and methods

The methods described herein are similar or identical to those we have previously published.[20,27,30,31,32,33,34,35]

### 2.1. Study subjects

The study cohort has been described previously; in brief, the cohort was a large population-based sample consisting of three separate recruitment efforts totaling 1,174 healthy children and younger adults (age 11 to 22 years) from all socioeconomic strata in Olmsted county, MN. [31] Of these, 1,101 participants had written records of having received two doses of MMR II vaccine (Merck; each dose containing not less than 1,000 TCID_50_ of the Wistar RA 27/3-strain of rubella virus) and agreed to participate in the study. One-hundred-fifty study participants with available sample, representing the top and the bottom of the rubella-specific neutralizing Ab responses (75 high Ab responders with a median titer of 219.1 NT_50_, and 75 low responders with a median titer of 22.4 NT_50_), were selected from this cohort for microarray profiling of rubella-specific humoral immunity. The Mayo Clinic Institutional Review Board approved the study. Written informed consent was obtained from each adult subject, and from the parents of all children who participated in the study.

### 2.2. Rubella neutralization assay

We have previously described the modified version of the Centers for Disease Control and Prevention (CDC) immuno-colorimetric-based rubella virus-specific neutralization assay, which was optimized by our laboratory to a high-throughput micro-format and used in large population-based studies. [20,27,30,31,32,33,34,35] Each assay contained the following controls: virus-only control (no serum); uninfected control (no serum or virus); and two reference sera (CDC anti-rubella human serum reference preparation IS2153 and a seronegative serum RP-011 panel member 1 [Biomex GmbH; Heidelberg, Germany]). The neutralization titer was calculated as the highest dilution at which the input virus signal was reduced by at least 50% within the dilution series (NT_50_). The intra-class correlation coefficient (ICC) based on log-transformed estimates from repeated NT_50_ measurements was 0.89, which demonstrates a high degree of reproducibility.

### 2.3. Pathogen array

We used the proteome microarray chips (Antigen Discovery, Inc., Irvine, CA) developed by PCR amplification of cDNA for all rubella virus proteins, as previously described. [24,25,26,27] Briefly, the amplicons were inserted into pXi T7-based exvectors, expressed in coupled *in-vitro* transcription-translation (IVTT) reactions, and printed onto microarray slides as protein/polypeptide spots representing the individual rubella virus proteins/polypeptides. Serum samples were diluted 1:100 in Protein Array Blocking Buffer (Whatman, Inc.; Sanford, ME) supplemented with 10% DH5-α *Escherichia coli* lysate (Antigen Discovery, Inc.), incubated for 30 minutes, and probed on arrays overnight at 4°C. The next day, microarray slides were incubated in Fc-specific Biotin-SP-Conjugated Affini-Pure Goat Ant-Human IgG secondary Ab (Jackson ImmunoResearch, Inc.; West Grove, PA). Bound antibodies were detected by incubation with streptavidin-conjugated SureLight® P3 (Columbia Biosciences; Columbia, MD). The array slides were scanned using a GenePix® 4300 Microarray Scanner (Molecular Devices; San Diego, CA) and quantified using GenePix® Pro 7 Microarray Acquisition and Analysis Software (Molecular Devices; Sunnyvale, CA) with spot-specific background correction. Due to the gene (protein) length of P150 and P90, they were amplified in segments overlapping by 150 nucleotides and expressed on the chip as three spots of overlapping polypeptides/fragments for P150 (i.e., P150s1, P150s2, and P150s3), and three spots for P90 (i.e., P90s1, P90s2, and the whole P90). [36] The capsid C protein and Glycoproteins E1 and E2 were expressed on the chip as single spots. All samples were run in triplicate against nine proteins/polypeptides (i.e., the above six polypeptides/proteins plus E1, E2, and C rubella proteins), and the median values were calculated and normalized. Antibody reactivity to each rubella virus protein/polypeptide was considered positive when the intensity value was greater than the corresponding background intensity value (no DNA/no expressed protein control).

### 2.4. Rubella-specific secreted cytokines

Secreted cytokines were measured after rubella virus stimulation of PBMC cultures, as previously described. [30,34] Briefly, 2 x10^6^/ml PBMCs were stimulated with the W-Therien strain of rubella virus (a gift from Dr. Teryl Frey, Georgia State University; Atlanta, GA) with optimized multiplicity of infection and incubation times (MOI of 5, 24h for IL-6 and MOI of 5, 48h for IFNγ). Secreted cytokines in supernatants were quantified using BD OptEIA™ Human ELISA kits. Absorbance levels were measured using a Molecular Devices SpectraMax 340PC.

### 2.5. Statistical analyses

As described previously, Normalization of the microarray reactivity was done by dividing the median antibody reactivity (signal intensity) for each protein by the median intensity of the ‘no DNA’(no expressed protein) controls. [27] Normalized results are presented on the log2 scale, and all analyses are done using the log2 of the normalized values. [27] Wilcoxon rank sum tests were used to test for statistically significant differences between the high and low Ab response groups in continuous variables, including rubella virus protein Ab measurements. Differences in sex and race in Ab response groups were tested with Pearson’s chi- square test. Spearman’s non-parametric correlation was used to assess the relationship between the protein Ab measurements and rubella-specific neutralizing antibody titers and secreted cytokines. A multivariate penalized logistic model was constructed using elastic-net regression. Specifically we used the “glmnet” package in the R-statistical software with α = 0.90, and the penalization parameter λ, selected at the minimum misclassification error after 10-fold cross validation.[37] Results are presented as standardized coefficients, and model efficiency is evaluated with the misclassification error and Brier’s score. [38]

## Results and discussion

The proposed correlate of protective immunity for rubella is a rubella-specific Ab titer of 10–15 international units per milliliter (IU/mL), corresponding to a neutralization Ab titer of 1:8. [39,40,41] The measurement of rubella-specific antibodies in clinical settings is generally performed using quantitative commercial immunoassays (including automated analytical systems based on immunofluorescence, electrochemiluminiscence, chemiluminiscence, etc.), which report the results in IU/mL using the currently available WHO international reference rubella standard. The lack of appropriate calibration of the international standard and standardization of the commercial IgG rubella virus-specific assays leads to inconsistencies in reporting (i.e. rubella-immune vs. rubella non-immune), particularly for samples with Ab concentration on the lower end of the spectrum. [1,6,21,22,23] Immunoblot and neutralization assays are regularly used as valuable reference assays for assessment of functional rubella humoral immunity and confirmation of equivocal and/or negative results. [21,22] We have previously used a high-throughput colorimetric neutralization assay to measure rubella virus-specific neutralizing antibodies in more than 1,000 samples in a large population-based study. [20,31,32,33,34] To evaluate the ability of a new rubella virus-specific antigen microarray chip to measure antibodies to different rubella virus proteins relevant to neutralizing Ab response, we tested sera from subjects at the extremes of the rubella-specific neutralizing Ab titer in our population-based study (i.e., 75 high Ab responders [high Ab group] and 75 low responders [low Ab group] selected for the current study) following two doses of MMR-II vaccine (Merck), containing the Wistar RA 27/3-strain of rubella virus.

The demographic and clinical variables of the study subjects are summarized in Table 1. Statistically significant differences in vaccine history variables were found between the two vaccine responder groups: the low Ab responders received their second MMR dose earlier and had a longer time interval from second vaccination to enrollment (blood draw) than the high Ab responders (Table 1). In line with these findings, previous studies have demonstrated similar associations between rubella vaccine immune outcomes (including vaccine-induced Ab titer) and vaccine history variables. [42,43,44,45]

**Table 1:** Demographic and clinical variables of the study cohort.

Rubella Study Subjects’ Demographics				
	High Ab Group (n = 75)	Low Ab Group (n = 75)	Total (n = 150)	p value^2^^,^^3^
**Age at enrollment (years)**				0.34^2^
Median (IQR^1^)	15.0 (13.0, 17.0)	15.0 (13.0, 17.0)	15.0 (13.0, 17.0)	
**Age at first vaccination (months)**				0.06^2^
Median (IQR^1^)	15.0 (15.0, 24.0)	15.0 (15.0, 16.0)	15.0 (15.0, 17.0)	
**Age at second vaccination (years)**				<0.0001^2^
Median (IQR^1^)	11.0 (6.0, 12.0)	7.0 (4.0, 11.0)	9.0 (5.0, 12.0)	
**Time from second vaccination to** **enrollment (years)**				<0.0001^2^
Median (IQR^1^)	5.5 (3.0, 7.4)	7.5 (5.6, 9.5)	6.4 (4.4, 8.5)	
**Gender**				0.74^3^
Male	45 (60.0%)	43 (57.3%)	88 (58.7%)	
Female	30 (40.0%)	32 (42.7%)	62 (41.3%)	
**Race**				0.0001^3^
Black/African American	18 (24.0%)	1 (1.3%)	19 (12.7%)	
White	55 (73.3%)	67 (89.3%)	122 (81.3%)	
Other	2 (2.7%)	7 (9.3%)	9 (6.0%)	

^1^IQR,25% and 75% inter-quartile range

^2^P-values are calculated using the Kruskal-Wallis test

^3^P-values are calculated using the Wilcoxon Rank Sum test

The new microarray chip allowed the detection of antibodies to all rubella virus structural proteins (i.e., the glycoproteins E1 and E2 and the capsid C protein, see Table 2), although anti-E2 antibodies were detected in only 15.3% of the study subjects (in 28% of the subjects in the high Ab responder group and 2.67% of the subjects in the low Ab responder group). This is not unexpected given the reported dynamics of humoral immunity to this specific protein since anti-E2 antibodies appear later compared to anti-E1 antibodies, and may be absent several months-to-years post-vaccination/infection. [6,46,47] Antibodies to three proteins/polypeptides (i.e., P90, P90s2, and P150s3), for which positive normalized values were measured in < 10% of the tested individuals, were considered undetectable (by the microarray technology) and removed from further analysis (Table 2). The two non-structural rubella virus proteins P150 and P90 are expressed only in infected cells, are required for viral replication, and are generally considered non-immunogenic. [6] Nevertheless, our data indicate that a percentage of vaccine recipients do develop humoral responses targeting epitopes located in the nonstructural P150 protein (i.e., P150s1 and P150s2, but not the P150s3 portion, Table 2). The relevance of these antibodies as markers of rubella vaccine-induced immunity and/or disease is yet to be determined.

**Table 2:** Characterization of the rubella-specific humoral immune response to different rubella virus proteins in the study cohort.

Protein	High Ab / Median^1^, IQR	N / % positive	Low Ab / Median^1^, IQR	N / % positive	All Subjects / Median^1^, IQR	N / % positive	Median Difference^2^	p-value^3^
RV.E2	-0.42 (-1.02, 0.09)	21/28.0	-1.39 (-1.77, -0.93)	2/2.7	-0.97 (-1.5, -0.31)	23/15.3	0.97	**9.45E**^**-12**^
RV.CP	0.88 (0.5, 1.26)	70/93.3	0.28 (-0.01, 0.65)	56/74.7	0.59 (0.16, 0.96)	126/84.0	0.61	**3.15E**^**-08**^
RV.E1	1.21 (0.57, 1.74)	69/92.0	0.27 (-0.27, 0.77)	49/65.3	0.73 (0.11, 1.47)	118/78.7	0.94	**3.22E**^**-08**^
RV.P150.s2	0.28 (-0.15, 0.53)	52/69.3	0.03 (-0.29, 0.31)	41/54.7	0.16 (-0.24, 0.47)	93/62.0	0.25	**0.008**
RV.P90.s2	-0.92 (-1.27, -0.66)	5/6.7	-1.09 (-1.38, -0.62)	4/5.3	-0.99 (-1.32, -0.64)	9/6.0	0.17	0.099
RV.P90	-0.90 (-1.09, -0.45)	8/10.7	-0.97 (-1.16, -0.53)	5/6.7	-0.90 (-1.13, -0.49)	13/8.7	0.08	0.187
RV.P150.s3	-2.11 (-2.35, -1.77)	0/0.0	-2.11 (-2.59, -1.79)	0/0.0	-2.11 (-2.43, -1.78)	0/0.0	0.003	0.271
RV.P150.s1	-0.04 (-0.25, 0.52)	35/46.7	0.04 (-0.5, 0.45)	41/54.7	0.01(-0.39, 0.52)	76/50.7	-0.08	0.367
RV.P90.s1	-0.80 (-1.25, -0.21)	15/20.0	-0.73 (-1.25, -0.36)	9/12.0	-0.77 (-1.26, -0.27)	24/16.0	-0.06	0.666

^1^Represents the median intensity of triplicate measurements (log_2_ of normalized value) for Ab reactivity against each rubella virus protein with the 25% and 75% inter-quartile ranges (IQR). RV designates rubella virus. The number/percentage of individuals with positive response to this antigen is also presented for each group.

^2^Represents the median difference between the Ab reactivity for a specific rubella virus protein (log_2_ of normalized value) in the high Ab responder group and the low Ab responder group

^3^P-values (for difference between the measurements in the high Ab and the low Ab group) were calculated using Wilcoxon rank sum test (p-values <0.05 are bolded)

**Fig 1:**
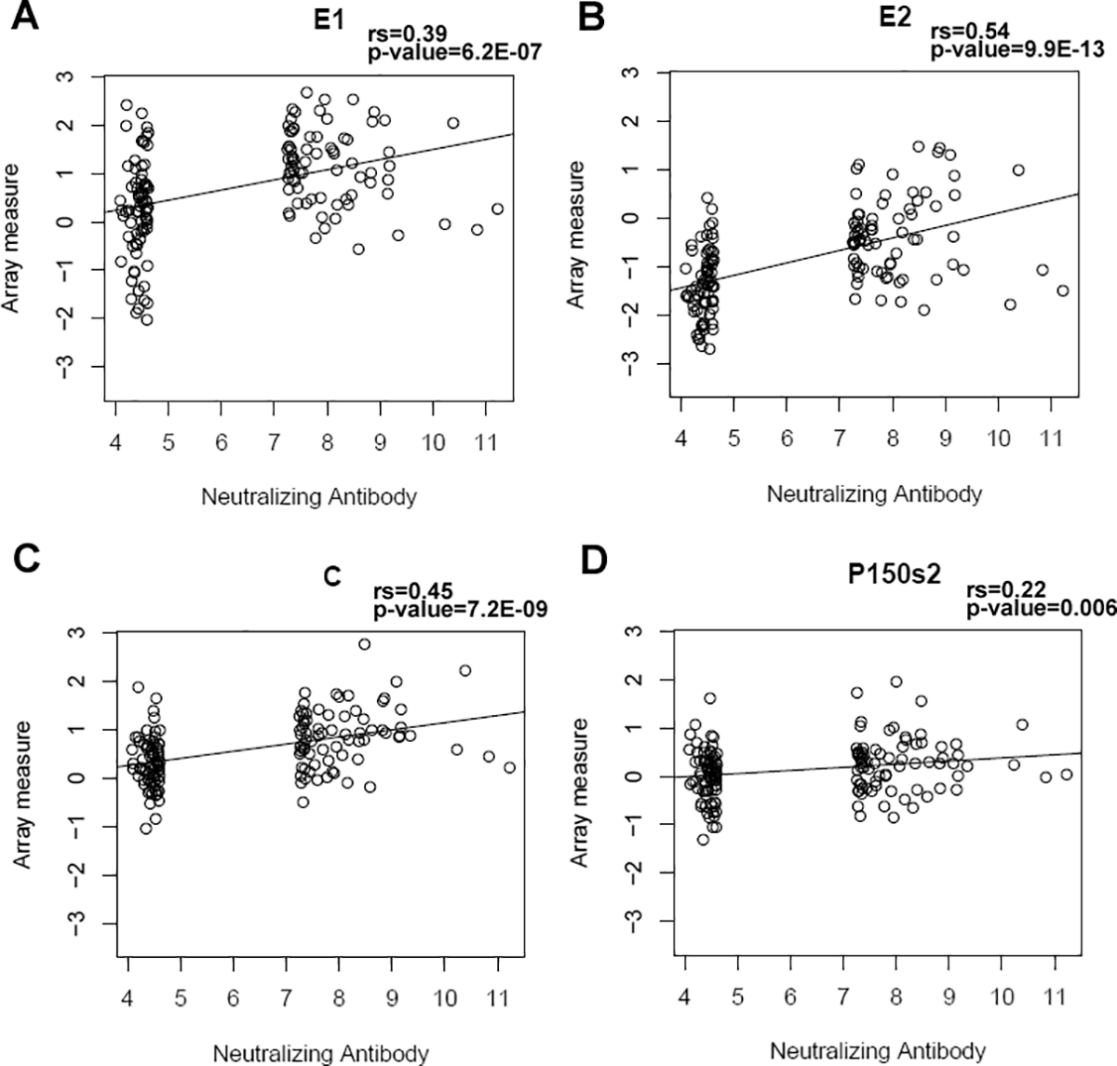
Correlation between microarray Ab measurements and rubella virus-specific neutralizing Ab titers after two MMR vaccinations. Panels A, B, C and D demonstrate the positive correlations (using Spearman’s correlation to test for significant relationships) between microarray Ab reactivities (on the y-axis, presented as log_2_ normalized signal intensity measures) against rubella virus E1, E2, C and P150s2 proteins, respectively, and neutralizing Ab response (presented as log_2_ value of the NT_50_ titer). “rs” indicates Spearman’s correlation coefficient.

As anticipated, statistically significant differences were observed between the signal intensity values for E1 (antibodies against E1 glycoprotein) of the sera of high Ab responders compared to low Ab responders (p-value = 3.22E^-08^), as well as for E2 (p-value = 9.45E^-12^) and C (p-value = 3.15E^-08^) proteins (Table 2). In addition, subjects in the high Ab responder group also had higher anti-P150s2 antibodies compared to subjects in the low Ab responder group (p = 0.008, Table 2). In concert with this, we observed weak to moderate correlations between Ab reactivity to the same four proteins (E1, E2, C and P150s2) and rubella-specific neutralizing Ab titers (Fig 1). When the analysis was restricted to the high Ab responder group only, the correlations were not significant (S1 Fig). Correlations with other rubella virus-specific immune outcomes (secreted IL-6 and IFNγ) were not found, with the exception of a weak correlation between anti-C Ab reactivity and IFNγ production (p = 0.01).

Antibodies to the structural proteins—specifically to the surface glycoproteins E1 and E2—are expected to be associated with neutralizing Ab response (as found in our study), since E1 and E2 contain the known neutralization epitopes/specificities. [48] Our study also demonstrates a previously unknown association between antibodies directed against non-structural rubella virus proteins (i.e., P150) and neutralizing Ab response. In concert with this finding, studies of other single-stranded positive polarity RNA viruses (West Nile, yellow fever, dengue, and tick-borne encephalitis viruses) have presented evidence for the protective role of antibodies to viral non-structural proteins in animal models, hence the assessment of humoral immunity to these proteins should be considered in systems biology, seroprevalence and/or other vaccine studies. [28,49,50,51,52]

**Fig 2:**
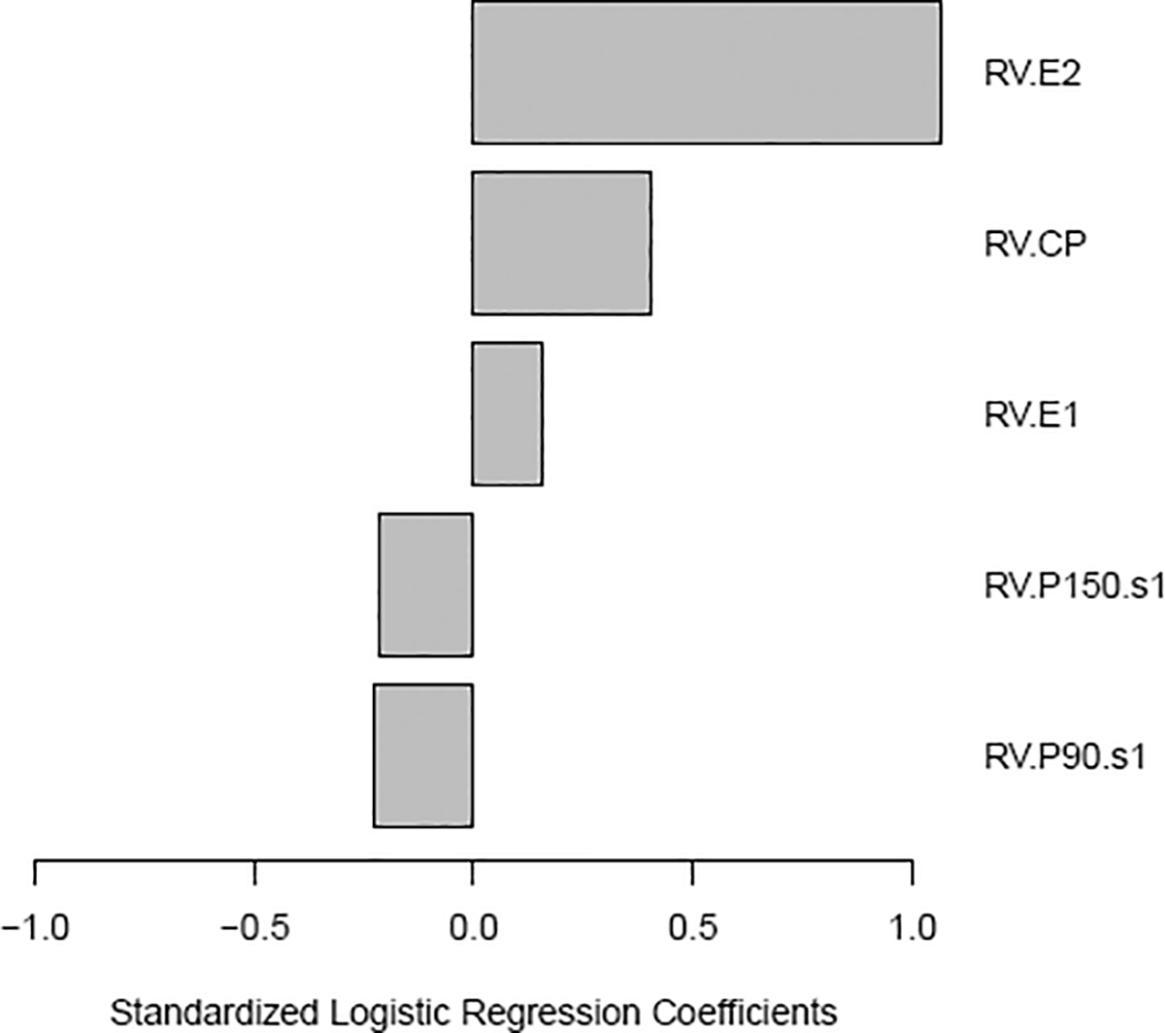
Logistic regression modeling of rubella-specific neutralizing Ab response after vaccination. Results from the elastic net logistic regression models for the association of the microarray Ab reactivities (to rubella virus proteins) with rubella virus-specific neutralizing Ab response. The modeling excluded antibodies to P90, P90s2 and P150s3, which were considered undetectable (had positive normalized values measured in < 10% of the tested subjects). The standardized logistic regression coefficients were as follows: 1.07 for anti-E2 Ab reactivity; 0.41 for anti-C Ab reactivity, 0.16 for anti-E1 Ab reactivity; -0.22 for anti-P150s1 Ab reactivity and -0.23 for anti-P90s1 Ab reactivity. The results are for the model with the misclassification error rate = 0.2, Brier Score = 0.15.

We applied multivariate penalized logistic regression modeling of rubella-specific neutralizing Ab response to identify Ab profiles associated with neutralization. Our model (Fig 2) points to the positive predictive value of antibodies against all three structural rubella virus proteins (with the highest relative contribution of anti-E2 antibodies) to neutralizing Ab response after rubella vaccination. Ab reactivity to domains in the non-structural proteins (P150s1 and P90s1) was negatively associated with high rubella-specific neutralizing Ab titer. In contrast to the immunodominant E1 glycoprotein, antibodies to E2 have limited neutralizing activity and E2 lacks the ability to elicit antibodies that inhibit hemagglutination. [7,8,9,10,11,12] While antibodies to E1 are functional in virus neutralization by hampering virus attachment/cell entry and E1 conformational changes/trimerization during cell entry, the neutralization mechanism of anti-E2 antibodies remains largely unknown. [48] Glycoprotein E2 function warrants additional studies, but this protein is likely involved in conformational changes during virus entry and maturation, E1 activation, E1 trafficking, and virus membrane budding. [48] Of interest, antibodies directed to E2 are more prevalent in individuals with CRS compared to vaccinations and non-CRS rubella infections, [53,54] and thus can be used, in combination with other antibodies, as a potential marker of rubella virus-specific pathology (CRS). An important limitation of our study is the inability of the microarray technology to measure humoral immunity against conformational antigenic epitopes (dependent on post-translational modification), which is also true for most of the rubella IgG assays. [27] As many of the rubella virus neutralizing epitopes are conformational, this could explain, in part, the moderate to weak correlations we observed (between the neutralizing antibodies and the antibodies directed to the surface rubella virus glycoproteins E1 and E2), as well as the weaker relative contribution of anti-E1 antibodies to neutralizing Ab response (representing functional protective immunity) in our statistical model. Our results and statistical model need further validation in an independent study, which is underway. The strengths of our study include the use of a robust new high-throughput technology and statistical modeling, which allow identification of humoral immune response profiles relevant to rubella virus neutralization. In addition to reactivity to different proteins (and/or polypeptides or epitopes), this method permits the identification of immunoglobulin classes (isotypes) and subclasses, and conceivably, avidity testing, which is important for the comprehensive assessment of immune response to primary (including CRS) and secondary rubella virus infections and immune response to vaccination.

In conclusion, the results of our study support the use of microarray technology and Ab profiles/patterns rather than single measures of humoral immunity to rubella virus protein/proteins (or whole virus) in rubella virus serology. The identified profiles can be used as useful biomarkers of rubella-specific neutralizing Ab response and protective immunity in systems biology, population genetics and/or other vaccine studies, while information on potential epitopes (Ab targets) can be applied in the rational design of new and/or improved rubella vaccines.

## Supporting information

S1 Fig Correlation between microarray Ab measurements and rubella virus-specific neutralizing Ab titers (after vaccination) in the high antibody responder group. (TIF)
