# Supplementary figures and images for: Characterization of rubella-specific humoral immunity following two doses of MMR vaccine using proteome microarray technology
Source: PLoS One. 2017 Nov 16;12(11):e0188149. doi: 10.1371/journal.pone.0188149 (PMC5690594; doi:10.1371/journal.pone.0188149)

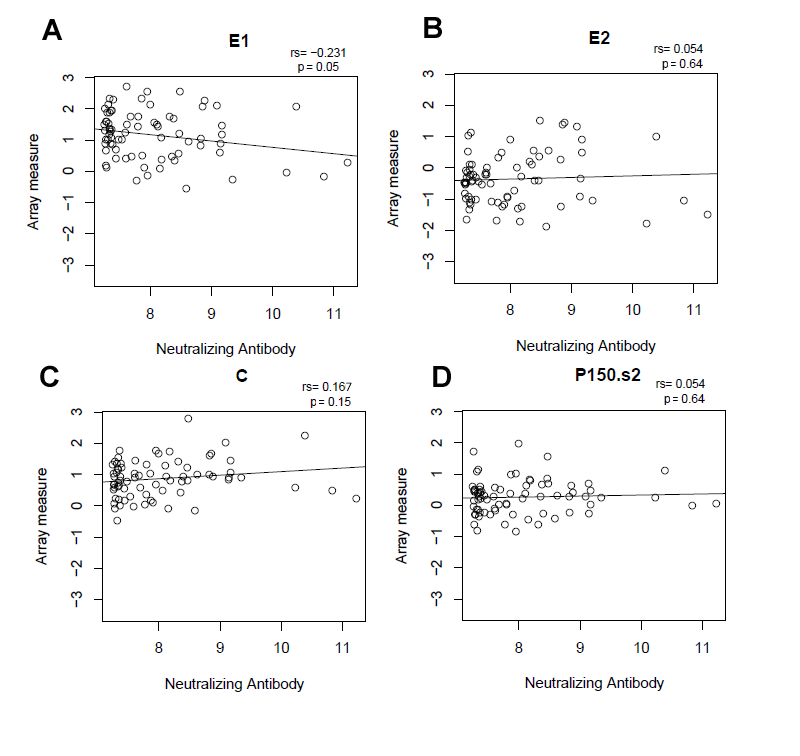

Supplement: S1 Fig — (TIF) [file pone.0188149.s001.tif]
